# UBE2T is a diagnostic and prognostic biomarker for endometrial cancer

**DOI:** 10.1007/s12094-024-03713-z

**Published:** 2024-10-05

**Authors:** Longyun Wang, Mengqi Wang, Zeyu Wang, Kai Wang, Bowei Zhao, Yue Wang, Jingying Zheng, Shuang Zhang

**Affiliations:** 1 Department of Rehabilitation, School of Nursing, Jilin University, Changchun, 130021 Jilin China; 2 Department of Reproductive Medicine, The Third Affiliated Hospital of Zhengzhou University, Zhengzhou, 450052 Henan China; 3 Department of Gynecology and Obstetrics, The Second Hospital of Jilin University, Changchun, 130000 Jilin China

**Keywords:** UBE2T, Bioinformatics, Diagnosis, Prognosis, Endometrial cancer

## Abstract

**Background:**

Endometrial cancer (UCEC) is one of the most common malignant tumors in gynecology, and early diagnosis is crucial for its treatment. Currently, there is a lack of early screening tests specific to UCEC, and treatment advances are limited. It is crucial to identify more sensitive biomarkers for screening, diagnosis, and predicting UCEC. Previous studies have shown that UBE2T is involved in the development of various tumors such as breast cancer and liver cancer, but research on the role of UBE2T in UCEC is limited.

**Methods:**

Using data from The Cancer Genome Atlas (TCGA), Gene Expression Omnibus (GEO), and UALCAN databases, we analyzed the differential expression of UBE2T mRNA and protein in endometrial cancer (UCEC), along with its clinical relevance. A total of 113 clinical samples were collected, and immunohistochemistry and Western blot analysis were employed to validate bioinformatics analysis results. Volcano plots were generated using UBE2T and its differentially expressed genes, and a protein–protein interaction (PPI) network was constructed. Gene Ontology (GO), Kyoto Encyclopedia of Genes and Genomes (KEGG), gene set enrichment analysis (GSEA), and immune infiltration analysis were used to predict the functional role of UBE2T in UCEC progression. Correlation between UBE2T expression and patient survival was analyzed using TCGA data, and Kaplan–Meier survival curves were plotted.

**Results:**

UBE2T is significantly overexpressed in UCEC and correlates with poor prognosis. Its overexpression is closely associated with mitosis, cell cycle regulation, and histological grade in UCEC patients.

**Conclusion:**

UBE2T is highly expressed in UCEC and suppresses anti-tumor immune responses in UCEC patients. It serves as a key participant in UCEC progression, associated with a range of adverse outcomes, and holds potential as a clinical diagnostic and prognostic biomarker.

## Introduction

Endometrial cancer (UCEC) is one of the most common tumors in the female reproductive system, second only to cervical cancer, ranking second among malignant tumors in the female reproductive system [1, 2]. Currently, treatment options for endometrial cancer are limited. For early-stage UCEC, surgery is typically performed first, followed by adjuvant therapy based on high-risk factors, achieving a 5-year survival rate of up to 90% [3]. Currently, treatment options for endometrial cancer are limited. For early-stage UCEC, surgery is typically performed first, followed by adjuvant therapy based on high-risk factors, achieving a 5-year survival rate of up to 90% [4]. Therefore, early diagnosis is a key factor in improving the prognosis of endometrial cancer patients, and exploring new sensitive biomarkers for early screening and diagnosis of UCEC is crucial [5].

Ubiquitin-conjugating enzyme E2T (UBE2T), also known as HSPC150 or Fanconi Anemia Group T protein (FANCT), is a component of the ubiquitin–proteasome degradation system. UBE2T was initially identified for its role in ubiquitinating FANCL, FANCD2, and FANCI, which are involved in DNA damage repair in Fanconi anemia. These proteins also play crucial roles in cell cycle progression, signal transduction, and tumorigenesis [6]. In recent years, UBE2T has been found to participate in the development of cancers such as liver cancer, breast cancer, and others [7–9]. UBE2T overexpression may downregulate BRCA1 expression, promoting breast cancer development [10]. High expression of UBE2T in bladder cancer is associated with enhanced proliferation and colony formation ability of bladder cancer cells when UBE2T is depleted [11]. UBE2T is also upregulated in hepatocellular carcinoma and promotes oncogenesis through p53 ubiquitination [12]. These studies suggest that UBE2T plays a critical role in the occurrence, growth, and progression of various malignancies.

However, there is currently insufficient research to establish the involvement of UBE2T in UCEC development. This study aims to further validate differential UBE2T gene expression in UCEC using the latest RNAseq data from TCGA, independently verify its expression using GEO database, and validate expression through immunohistochemistry in 113 clinical UCEC specimens. Specific functions of UBE2T in UCEC development will be explored using protein–protein interaction (PPI) networks, Gene Ontology (GO) term analysis, Kyoto Encyclopedia of Genes and Genomes (KEGG) pathway analysis, gene set enrichment analysis (GSEA), single-sample gene set enrichment analysis (ssGSEA), and Kaplan–Meier survival analysis to predict the role of UBE2T in UCEC patient prognosis.

## Materials and methods

### Data source and preprocessing

The differential RNAseq expression data of UBE2T in pancancer were obtained from UCSC XENA (https://xenabrowser.net/datapages/) in the TPM (transcripts per million reads) format of the TCGA and GTEx processed uniformly by the Toil process to ensure data consistency and comparability [13]. Additionally, RNAseq expression data of UBE2T in unpaired and paired samples were obtained in FPKM (Fragments Per Kilobase per Million) format from the TCGA (https://portal.gdc.cancer.gov/) UCEC project. These FPKM values were converted to TPM format and log2 transformed for comparison. All final analyses were performed using data in TPM format. The differential analysis data for UBE2T in dataset GSE17025, were downloaded from the GEO database using the GEOquery package (version 2.54.1) [14, 15]. These data were obtained by removing probes corresponding to multiple molecules, and when probes corresponding to the same molecule were encountered, only the probe with the largest signal value was retained, and then the data were normalized again by the normalize Between Arrays function of the limma package (version 3.42.2) [16]. All statistical analyses and visualizations were performed using R (version 3.6.3).

### Study population

This retrospective study was approved by the Ethics Committee of the School of Nursing, Jilin University (Changchun, China), and is in line with the principles of the Declaration of Helsinki. All enrolled patients were informed, agreed to participate in the study, and given written informed consent. Paraffin-embedded specimens from 113 female patients diagnosed with UCEC between January 1, 2019, and May 31, 2019, were obtained from the Second Hospital of Jilin University (Changchun, China). The inclusion criteria were as follows: the first operation was performed in the Second Hospital of Jilin University, and the pathological diagnosis was UCEC. The exclusion criteria were: other malignant tumors; use of an intrauterine device (IUD) or hormone therapy within 6 months before surgery. Between May 2021 and June 2022, 11 specimens were collected from freshly frozen tumors and adjacent non-cancerous tissues of UCEC patients. Baseline characteristics and pathological data, including age, menopausal status, degree of differentiation and FIGO stage, were extracted from Second Hospital of Jilin University database.

### Western blotting

Proteins were extracted from fresh-frozen tissues followed by protein quantitation with a Coomassie Plus (Bradford) Assay Kit (Thermo Scientific, Cat#23,236). Western blot analysis was conducted under standard procedures as previously described. The primary antibodies were UBE2T (Proteintech, Cat# 10,105–2-AP; Dilution, 1:2000) and GAPDH (Proteintech, Cat#10,494–1-AP; Dilution, 1:5000). The secondary antibody was HRP-conjugated goat anti-rabbit (Proteintech, Cat#SA00001-2; Dilution, 1:20,000).

### Immunohistochemistry

Immunohistochemical (IHC) staining assays were performed as previously described. Briefly, the paraffin-embedded tissues were cut into four μm thick slides. After deparaffinization and rehydration, antigen retrieval was performed with each slide. Then, slides were blocked with 5% serum and incubated with primary antibodies against UBE2T (Proteintech, Cat# 10,105–2-AP; Dilution, 1:200) overnight at 4 °C. The following procedure was performed: incubation with secondary antibodies, signal detection with DAB chromogen solution, counterstaining with haematoxylin, dehydration, and sealing with neutral gum. Finally, the slides were imaged using an Olympus optical microscope (BX51). The evaluation criteria were based on staining intensity and the proportion of positive tumor cells as previously described. For staining intensity: 0 (no colour), 1 (light yellow), 2 (yellow–brown), and 3 (brown). For the proportion of positive tumor cells: 0 (< 5%), 1 (5–25%), 2 (26–50%), 3 (51–75%), and 4 (> 75%). The scoring was evaluated by two independent pathologists blinded to the patients’ UCEC status [17].

### Differential expression analysis of UBE2T

The expression profile of UBE2T in different cancers was analyzed using the Mann–Whitney *U* test (Wilcoxon rank sum test). The Shapiro–Wilk normality test was used to examine the normality of UBE2T expression data in paired, unpaired, and GSE17025. The independent sample *t*-test was used to analyze the difference of the data in the unmatched samples, the paired sample *t*-test was used to analyze the difference between the paired samples, and the Mann–Whitney *U* test (Wilcoxon rank sum test) was used to analyze the variance of the data in GSE17025. All the above analysis results were visualized using ggplot2 (version 3.3.3) and were considered statistically significant when *p* < 0.05 [18].

### Single gene difference analysis and correlation analysis of UBE2T

The DESeq2 software package (version 1.26.0) was used to analyze the single gene difference of RNAseq data in UCEC (Endometrial cancer) tertiary HTSeq-Counts format in TCGA [19]. Single gene correlation analysis of expression profile data in TPM format was performed using the STAT software package (version 3.6.3). The target molecule of the above analysis is UBE2T. The single gene difference analysis results were used to draw the volcano map, and the threshold value |log2(FC)|> 1, p.dj < 0.05. These differentially expressed genes were recorded in the STRING database, and protein–protein interaction (PPI) of differentially expressed genes was analyzed using the Cytoscape network [20, 21]. Then, the MCODE plug-in was used to identify HUB genes. Finally, the single gene correlation analysis results were used to sort in descending order according to Pearson value, and the top 50 genes with correlation were extracted, and the single gene co-expression heat map of UBE2T was drawn using these genes and HUB genes. Use ggplot2 (version 3.3.3) to make volcano and co-expression heat maps.

### Functional enrichment analysis of UBE2T in UCEC

By using the clusterProfiler package (version 3.14.3), GO, KEGG and GSEA functional enrichment analysis were performed on the single gene difference analysis results [22]. Gene ID transformation was performed using the org.hs.egg.db package (version 3.10.0), and Z values were calculated using the GOplot package (version 1.0.2), which scores the correlation of UBE2T with enrichment pathways. The reference gene set of GSEA is c2.cgp.v7.2. Symbols. If they meet the conditions of false discovery rate (FDR) < 0.25 and p.adjust < 0.05, the results are significantly enriched. The above analysis results were visualized using ggplot2 (version 3.3.3).

### Immune infiltration analysis of UBE2T

The relative infiltration level of 24 immune cells was analyzed by the GSVA package (version 1.34.0) [23]. The immune infiltration algorithm was ssGSEA and Spearman was used for correlation analysis. Twenty-four immune cell markers were obtained from immune studies [24]. Subsequently, according to the expression of UBE2T, the samples were divided into low expression group and high expression group. The enrichment fraction of various immune cell infiltration in different subgroups was calculated, and the GSVA software package (version 1.34.0) was used for analysis. Finally, by analyzing immune cells with statistically significant relative infiltration (*p* < 0.001), the correlation between immune cell infiltration and UBE2T expression was visualized, and the UBE2T gene expression data was used to draw a string map. Statistical analysis and visualization using the circle software package (version 0.4.12).

### Statistical analysis

Data are expressed as mean ± standard deviation (mean ± SD). The difference in UBE2T expression between UCEC tumor tissue and adjacent tissue was analyzed by *t*-test. One-way analysis of variance (ANOVA) was performed to compare the differences among groups. The Mann–Whitney *U* test analyzed the correlation between UBE2T expression and clinical data of UCEC patients. Statistical plots were completed using GraphPad Prism 8, and *p* < 0.05 was considered statistically significant.

### Clinical correlation analysis and survival prognosis analysis

Survival data for UCEC patients were statistically analyzed using the survival package (version 3.2–10), and the results were visualized using the survminer package (version 0.4.9) to plot the overall survival (OS) and progression-free interval (PFI) of the Kaplan–Meier survival curve for UCEC patients [25]. We then performed a subgroup analysis of Kaplan–Meier survival curves for UCEC patients, analyzing clinicopathological factors such as age, presence of diabetes, and menopausal status. We then used these clinicopathological factors to calculate their association with UBE2T expression and visualized them using ggplot2 (version 3.3.3). ROC analysis of the data was performed using the pROC software package (version 1.17.0.1) to determine the accuracy of UBE2T in predicting prognosis.

## Results

### Differential expression of UBE2T in *pan*-*cancer* and UCEC

**Fig. 1:**
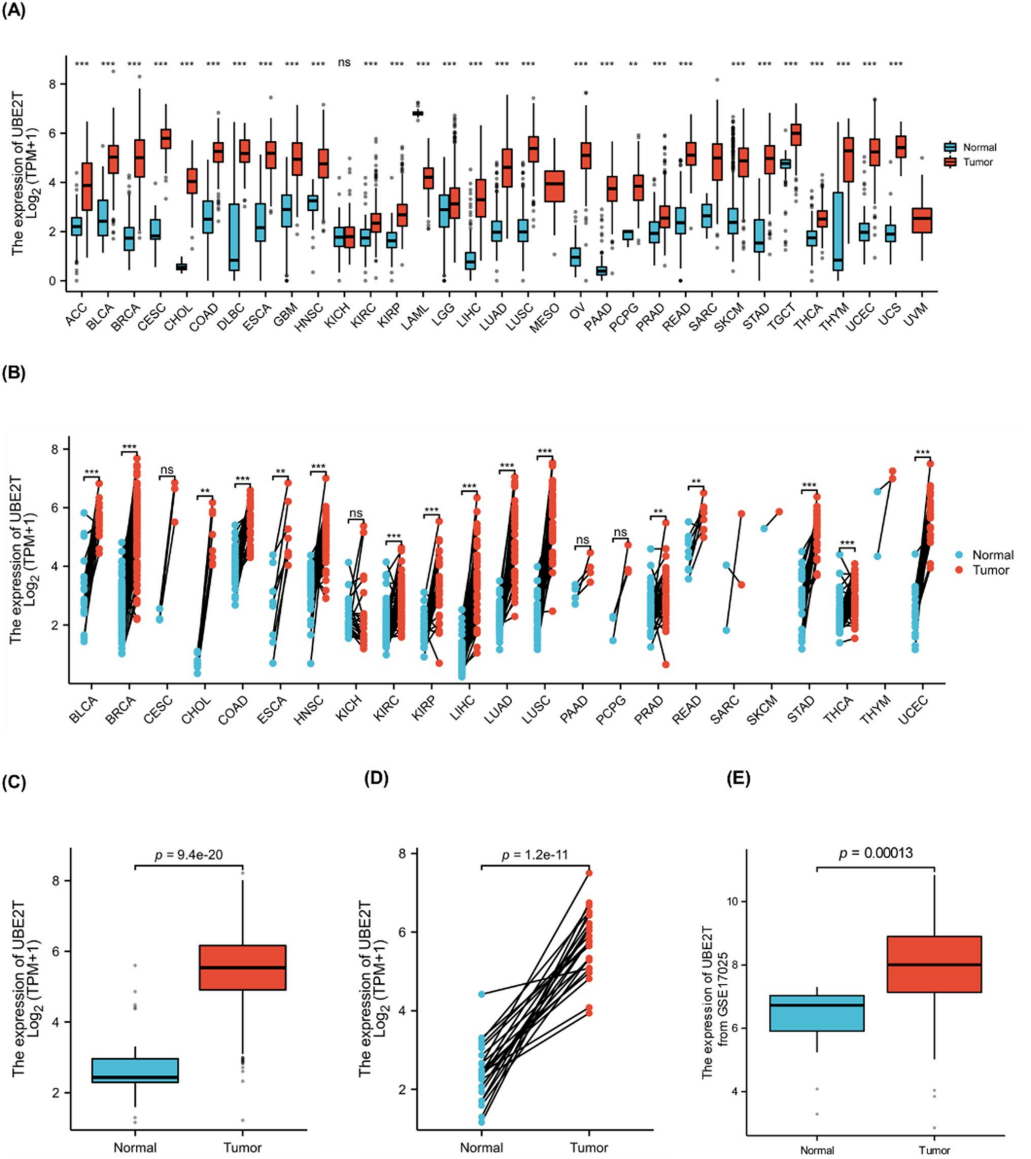
Differential expression of UBE2T in pan-cancer and UCEC. The data from the TCGA and GEO databases show that the expression level of UBE2T is higher in pan-cancer and UCEC. **A** Results of differential analysis of UBE2T expression in 33 tumors based on the data in the TCGA database. **B** Differential analysis of UBE2T expression based on paired pan-cancer tumor data from the TCGA database. **C** The expression of UBE2T is higher in tumor tissues in unpaired samples. **D** The expression of UBE2T is higher in tumor tissues in paired samples. **E** UBE2T is highly expressed in tumors based on the data in dataset GSE17025

The results of the differential expression analysis of UBE2T among cancers are shown in Fig. 1A. Adrenal cortical carcinoma (ACC, T = 77, N = 128, *p* = 7.95e-22), transitional cell carcinoma of bladder (BLCA, T = 407,N = 28, *p* = 3.42e-15), invasive ductal carcinoma (IDC) of breast (BRCA, T = 1099, N = 292, *p* = 1.6e-147), squamous cell carcinoma of cervix and adenocarcinoma of cervix (CESC, T = 306, N = 13, p = 1.05e-09), cholangiocarcinoma (CHOL, T = 36, N = 9, *p* = 4.59e-06), colon cancer (COAD, T = 290, N = 349, *p* = 3.48e-99), diffuse large B-cell lymphoma (DLBC, T = 47, N = 444, *p* = 4.49e-16), esophageal carcinoma (ESCA, T = 182, N = 666, *p* = 5.6e-83), glioblastoma multiformis (GBM, T = 166, N = 1157, *p* = 1.92e-75), head and neck squamous cell carcinoma (HNSC, T = 520, N = 44, *p* = 5.24e-22), kidney Clear cell carcinoma (KIRC, T = 531, N = 100, *p* = 3.39e-19), renal papillary cell carcinoma (KIRP, T = 289, N = 60, *p* = 7.82e-23), acute myeloid leukemia (LAML, T = 173, N = 70, *p* = 3.06e-34), low-grade glioma of the brain (LGG, T = 523, N = 1152, *p* = 0.0098), liver hepatocellular carcinoma (LIHC, T = 371, N = 160, *p* = 4.64e-62), lung adenocarcinoma (LUAD, T = 515, N = 347, *p* = 8.75e-122), lung squamous cell carcinoma (LUSC, T = 498, N = 338, *p* = 1.12e-130), ovarian serous cystadenocarcinoma (OV, T = 427, N = 88, *p* = 6.27e-49), pancreatic cancer (PAAD, T = 179, N = 171, *p* = 2.67e-57), pheochromocytoma and paratransit Ganglioma (PCPG, T = 182, N = 3, *p* = 0.0036), prostate adenocarcinoma (PRAD, T = 496, N = 152, *p* = 2.6e-28), rectal adenocarcinoma (READ, T = 93, N = 318, *p* = 4.49e-45), cutaneous melanoma (SKCM, T = 469, N = 813, *p* = 1.11e-139), gastric adenocarcinoma (STAD, T = 414, N = 210, *p* = 1.23e-82), testicular germ cell tumor (TGCT, T = 154, N = 165, *p* = 1.19e-36), thyroid cancer (THCA, T = 512, N = 338, *p* = 1.93e-88), thymoma (THYM, T = 119, N = 446, *p* = 1.73e-30), endometrial cancer (UCEC, T = 181, N = 101, *p* = 6.77e-42) and uterine carcinosarcoma (UCS, T = 57, N = 78, *p* = 4.7e-23), the expression level of UBE2T was higher than that of normal tissues, and the difference was statistically significant (*p* < 0.05). As shown in Fig. 1B, in pan-cancer pairs, UBE2T is detected in BLCA(T = 19, N = 19, *p* = 1.91e-05), BRCA(T = 113, N = 113, *p* = 4.47e-20), CHOL(T = 8, N = 8, *p* = 0.0078), COAD(T = 41, N = 41, *p* = 6.37e-12), ESCA(T = 8, N = 8, *p* = 0.0078), HNSC(T = 43, N = 43, *p* = 5.75e-11), KIRC(T = 72, N = 72, *p* = 3.85e-10), KIRP (T = 32, N = 32, *p* = 5.87e-07), LIHC (T = 50, N = 50, *p* = 7.79e-10), LUAD (T = 58, N = 58, *p* = 3.6e-11), LUSC (T = 49, N = 49 *p* = 3.55e-15), PRAD (T = 52, N = 52, *p* = 0.0015), READ(T = 9, N = 9, *p* = 0.0078), STAD (T = 27, N = 27, *p* = 1.49e-08), THCA (T = 59, N = 59, *p* = 1.87e-07), UCEC (T = 23, N = 23, *p* = 2.38e-07) were higher than neighboring tissues, and the difference was statistically significant (*p* < 0.05).

In the unpaired and paired samples of UCEC, the expression of UBE2T was significantly different from that of normal samples, as shown in Fig. 1C (*p* = 9.4e-20), D (*p* = 1.2e-11).

Then, we used the difference analysis of the protein expression of UBE2T in the GEO database to verify the results in the TCGA database. The results are shown in Fig. 1E, and the results are still significantly different (*p* = 0.00013).

### Evaluation of the expression of UBE2T in clinical samples of UCEC

**Fig. 2:**
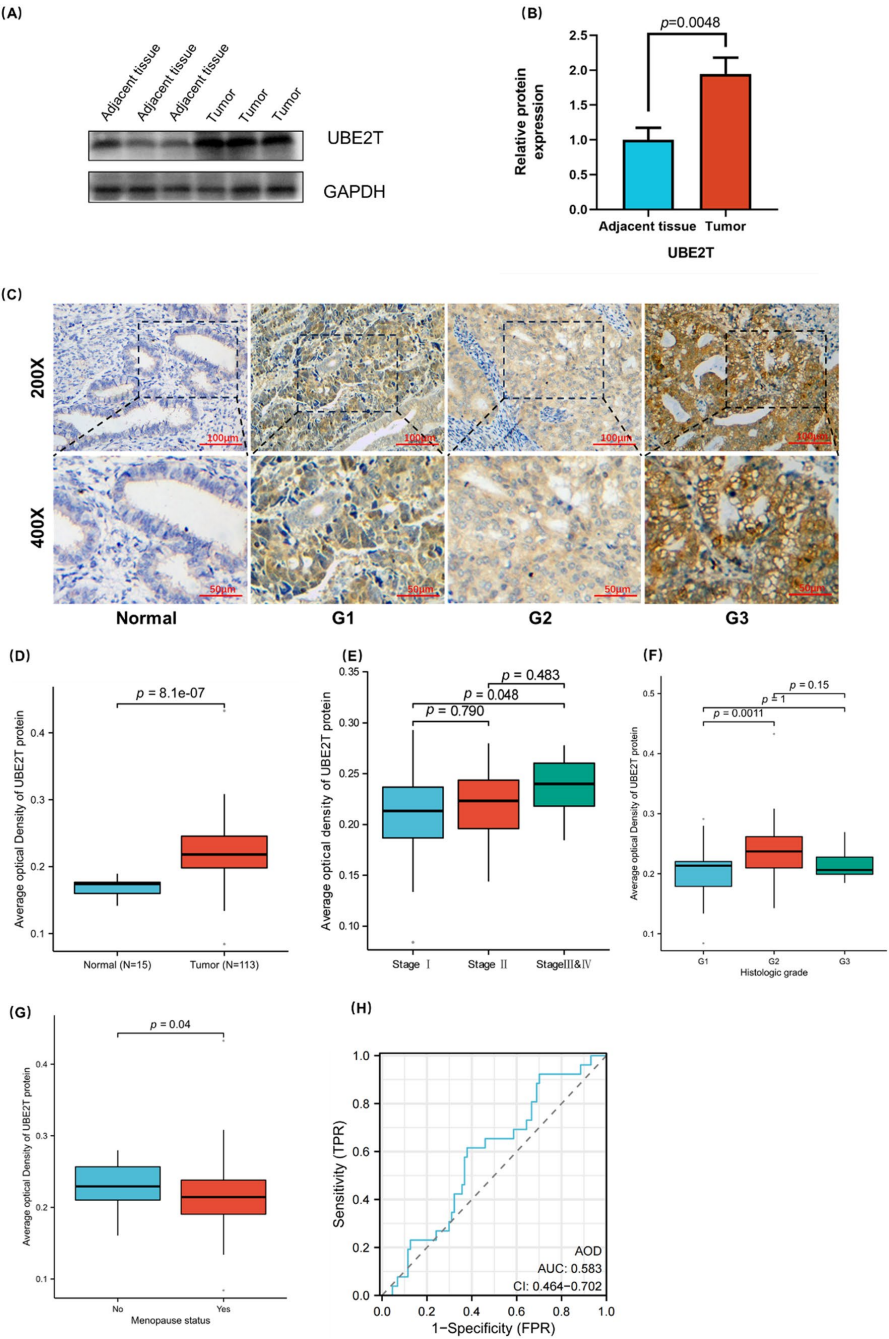
Expression and clinical correlation analysis of UBE2T in UCEC clinical samples. The expression level of UBE2T is higher in clinical samples of UCEC, and is associated with prognosis. **A**–**B** The expression of UBE2T in UCEC is higher than UCEC-adjacent tissues detected by Western blot analysis. **C** Immunohistochemical results of UBE2T in normal endometrial and UCEC tissues with different degrees of differentiation. **D** Group comparison of UBE2T immunohistochemical results in 113 UCEC clinical specimens and 15 normal endometrial cancer tissues. **E**–**G** Group comparison of UBE2T protein expression levels in samples with different clinical characteristics, **E** FIGO stage, **F** Histologic grade, and **G** Menopause status. **H** The diagnostic ROC curve of UBE2T

Next, we evaluated the expression of UBE2T in 10 UCEC tumor tissues and 10 adjacent tissues by Western blot analysis. As shown in Fig. 2A and B, the expression of UBE2T was higher in UCEC tumor tissues than in adjacent tissues (p= 0.0048). AOD was determined by immunohistochemical staining in 15 normal tissue samples and 113 UCEC samples. As shown in Fig. 2C, the expression of UBE2T is different in tissues with different degrees of differentiation. The expression level of UBE2T is lower in normal tissues, while the lower the degree of tumor differentiation, the higher the expression level of UBE2T. Figure 2D shows the expression level of UBE2T in 15 normal samples and 113 UCEC samples. There is a certain difference between the two, and the expression of UBE2T in tumor tissues is significantly increased (p= 8.1e-07). As shown in Fig. 2E–G, UBE2T expression levels were different in patients with different FIGO stages (Stage I vs Stage III & IV, p=0.048), histological grades (G1 vs G2, p= 0.0011), and menopause status (p= 0.04). The ROC curve of UBE2T protein expression data is shown in Figure H, with AUC=0.583, suggesting that UBE2T may be closely related to the occurrence and development of tumors.

### Single gene difference analysis and correlation analysis of UBE2T

**Fig. 3:**
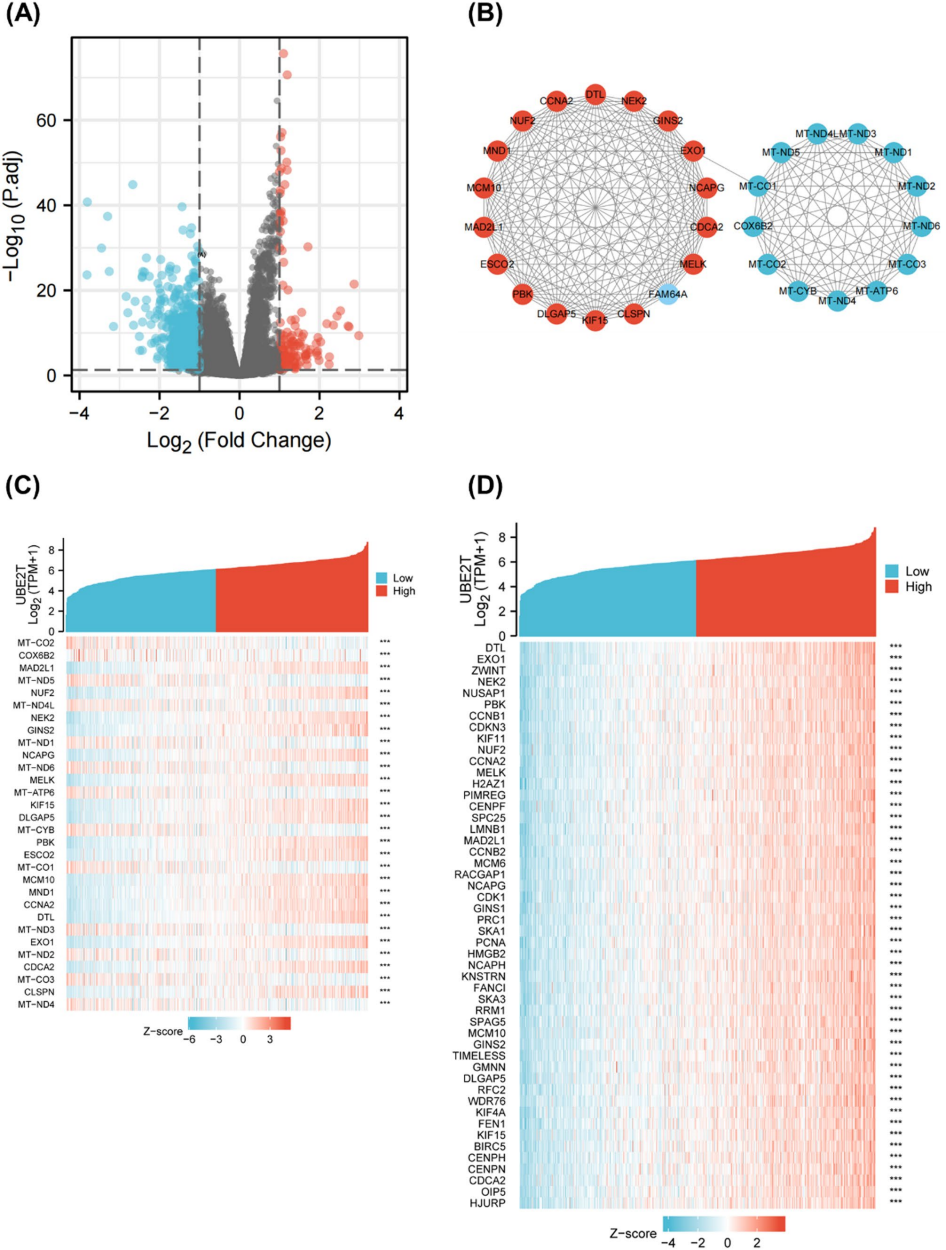
Single gene difference analysis and correlation analysis of UBE2T. Mitochondrial proteins showed a significant correlation with UBE2T expression. **A** Volcano plot of single gene differential analysis of UBE2T. **B** Protein–protein interaction network (PPI) of differentially expressed genes in single gene differential analysis. **C** Single gene co-expression heatmap of the HUB gene and UBE2T. **D** Single gene co-expression heatmap of the top 50 most strongly correlated genes with UBE2T in single gene correlation analysis

Single gene difference analysis for UBE2T was performed in UCEC, as shown in Fig. 3A. A total of 818 genes met the requirements of |log2 (FC) |> 1 and p.adj < 0.05. Under this threshold, 127 genes had high expression, and 691 genes had low expression. These 818 differentially expressed genes were used to construct the PPI network, and the results are shown in Fig. 3B. Genes located closer to the center of the interaction network have more connections with other genes. Using the MCODE plug-in, 30 HUB genes were identified. Namely MT-CO2, COX6B2, MAD2L1, MT-ND5, NUF2, MT-ND4L, NEK2, GINS2, MT-ND1, NCAPG, MT-ND6, MELK, MT-ATP6, KIF15, DLGAP5, MT-CYB, PBK, ESCO2, MT-C O1, MCM10, MND1, CCNA2, DTL, MT—ND3, EXO1, MT—ND2, CDCA2, MT—CO3, CLSPN, MTND4. The PPI network of the HUB gene is shown in Fig. 3C. These HUB genes are all related to cell mitosis. Based on these genes, we mapped their gene coexpression heat maps with UBE2T, as shown in Fig. 3C. Single gene correlation analysis was carried out for UBE2T, and the top 50 genes with the highest correlation were selected to draw the correlation heat map with UBE2T, and the results are shown in Fig. 3D.

### Functional enrichment analysis of UBE2T in UCEC

**Fig. 4:**
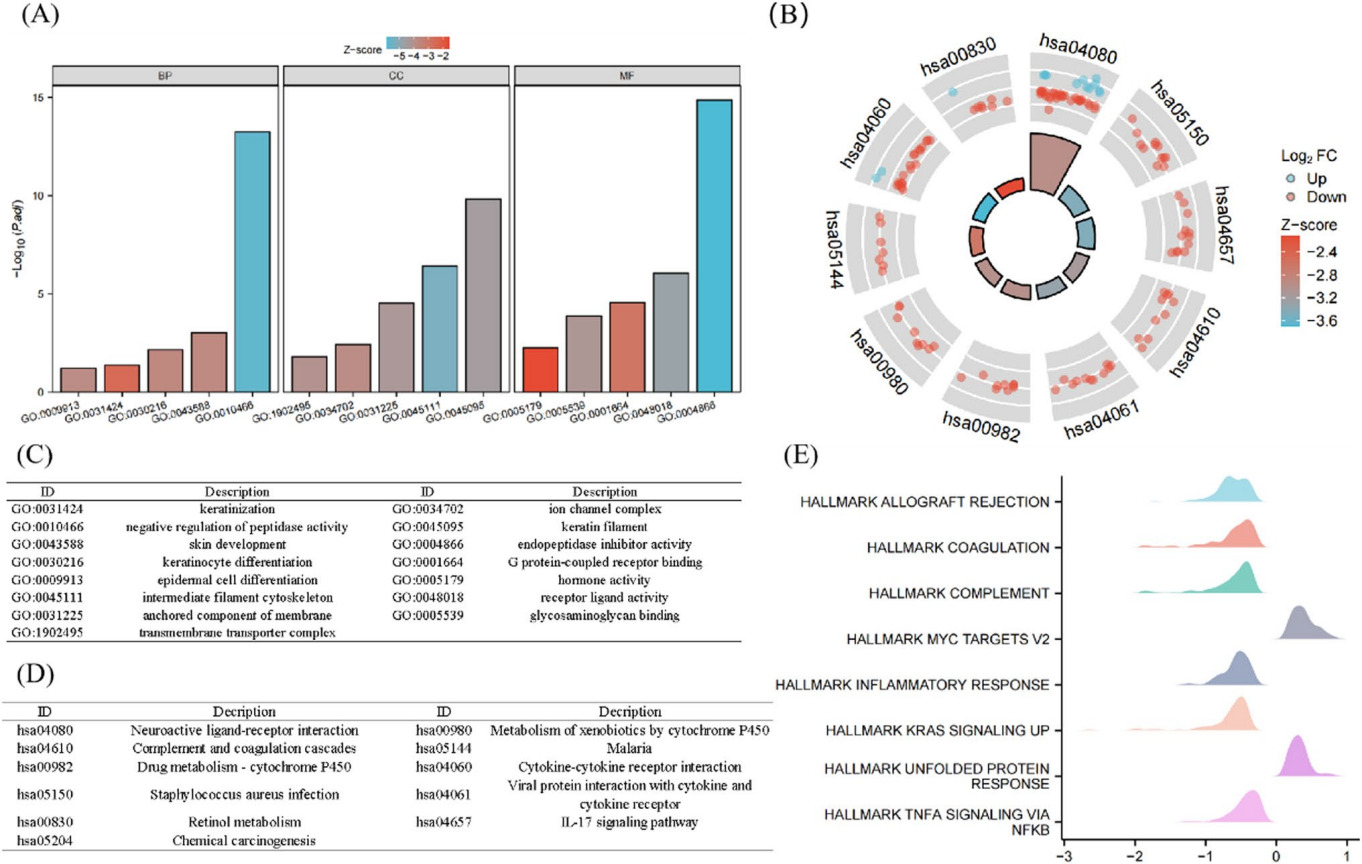
Functional enrichment analysis of UBE2T in UCEC. UBE2T is closely associated with tumor cell proliferation. (**A**) Results of GO analysis. **B** Results of KEGG analysis. **C**, **D** GO and KEGG analysis category names corresponding to GO and KEGG identifiers. **E** The results of GSEA. When the horizontal coordinate is positive, it suggests that the expression of UBE2T positively correlates with the pathway, while the opposite is true when the horizontal coordinate is negative

The results of single gene difference analysis were used for GO, KEGG and GESA enrichment analysis, and the results are shown in Fig. 4. Figure 4A, C, and Table 1 show the results of GO analysis, which show that UBE2T is functionally associated with keratinization, negative regulation of peptidase activity, skin development, keratinocyte differentiation, and keratin fibers. Figure 4B, D, and Table 1 show the results of KEGG analysis, indicating that UBE2T is associated with neuroactive ligand–receptor interaction, complement system, IL17 signaling pathway, chemical carcinogenesis, etc. The Z-score reflects the correlation between UBE2T and these pathways to some extent. A negative Z-score indicates a negative correlation, and a positive Z-score indicates a positive correlation. Figure 4E reflects the enrichment and grading results of GESA, which showed significant enrichment of tumorigenesis-related genes such as unfolded protein response, MYC Targets v2, upregulated KRAS signal, and NF-κB signaling pathway, suggesting that UBE2T was closely related to the tumor.

**Table 1:** Detailed results of GO and KEGG analysis

Ontology	ID	Description	Gene Ratio	Bg Ratio	*p*-value	p.adjust	*q* value
BP	GO:0031424	Keratinization	37/668	224/18670	6.05147E-15	3.95564E-12	3.70944E-12
BP	GO:0010466	Negative regulation of peptidase activity	44/668	262/18670	8.04924E-18	3.15691E-14	2.96043E-14
BP	GO:0043588	Skin development	52/668	419/18670	4.47584E-15	3.95564E-12	3.70944E-12
BP	GO:0030216	Keratinocyte differentiation	42/668	305/18670	6.02816E-14	3.37749E-11	3.16728E-11
BP	GO:0009913	Epidermal cell differentiation	43/668	358/18670	3.44681E-12	1.50204E-09	1.40855E-09
CC	GO:0045111	Intermediate filament cytoskeleton	33/726	251/19717	2.34606E-10	3.23756E-08	2.87289E-08
CC	GO:0031225	Anchored component of the membrane	22/726	170/19717	3.09652E-07	1.60245E-05	1.42196E-05
CC	GO:1,902,495	Transmembrane transporter complex	26/726	324/19717	0.000179052	0.004118203	0.003654342
CC	GO:0034702	Ion channel complex	24/726	301/19717	0.000343766	0.006294589	0.005585587
CC	GO:0045095	Keratin filament	23/726	95/19717	3.97856E-13	1.64712E-10	1.4616E-10
MF	GO:0004866	Endopeptidase inhibitor activity	37/652	175/17697	3.30943E-18	1.05074E-15	9.10964E-16
MF	GO:0001664	G protein-coupled receptor binding	30/652	280/17697	1.69809E-07	1.19809E-05	1.03871E-05
MF	GO:0005179	Hormone activity	14/652	122/17697	0.000160938	0.004836372	0.004192992
MF	GO:0048018	Receptor ligand activity	47/652	482/17697	1.22787E-09	1.11385E-07	9.65679E-08
KEGG	hsa04080	Neuroactive ligand–receptor interaction	37/278	341/8076	3.41267E-10	8.36104E-08	7.79525E-08
KEGG	hsa04610	Complement and coagulation cascades	10/278	85/8076	0.000635281	0.02451955	0.022860333
KEGG	hsa00982	Drug metabolism—cytochrome P450	9/278	71/8076	0.000682756	0.02451955	0.022860333
KEGG	hsa05150	Staphylococcus aureus infection	12/278	96/8076	0.000103128	0.008422145	0.007852225
KEGG	hsa00830	Retinol metabolism	8/278	68/8076	0.002195086	0.044816345	0.04178366
KEGG	hsa05204	Chemical carcinogenesis	9/278	82/8076	0.00193294	0.043051855	0.040138572
KEGG	hsa00980	Metabolism of xenobiotics by cytochrome P450	9/278	77/8076	0.001235654	0.033784822	0.031498631
KEGG	hsa05144	Malaria	7/278	50/8076	0.001490586	0.036519347	0.034048113
KEGG	hsa04060	Cytokine–cytokine receptor interaction	21/278	295/8076	0.001241075	0.033784822	0.031498631
KEGG	hsa04061	Viral protein interaction with cytokine and cytokine receptor	11/278	100/8076	0.000614602	0.02451955	0.022860333
KEGG	hsa04657	IL-17 signaling pathway	12/278	94/8076	8.38205E-05	0.008422145	0.007852225
KEGG	hsa04080	Neuroactive ligand–receptor interaction	37/278	341/8076	3.41267E-10	8.36104E-08	7.79525E-08
MF	GO:0005539	Glycosaminoglycan binding	25/652	229/17697	1.2771E-06	8.1096E-05	7.03078E-05

### Immune infiltration analysis of UBE2T

**Fig. 5:**
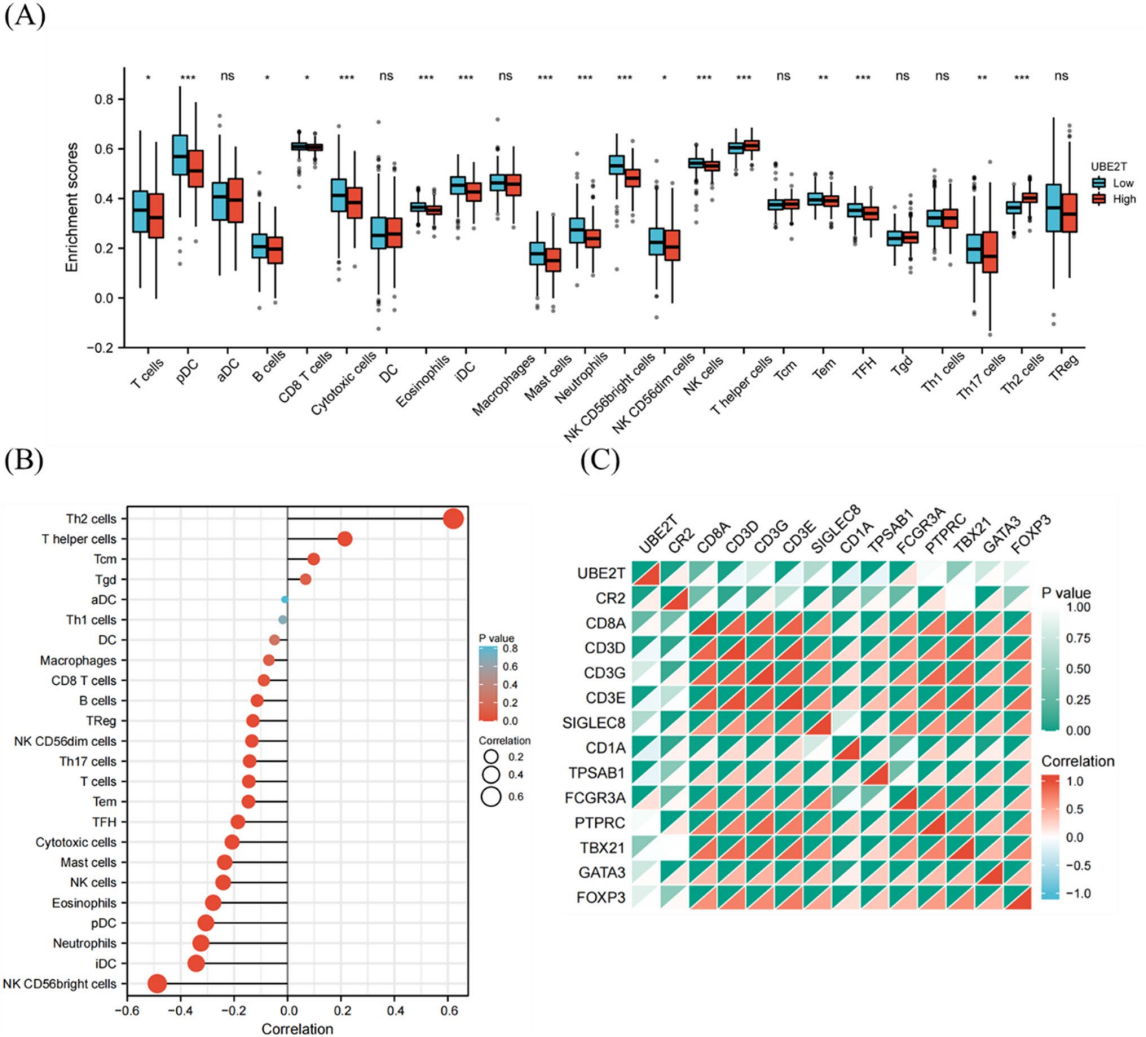
Immune infiltration analysis of UBE2T. The high UBE2T expression was negatively correlated with infiltration levels of most immune cells. **A** Grouped comparison of the infiltration levels of 24 immune cells in the low and high expression groups of UBE2T. **B** Results of ssGSEA of the correlation between the expression of UBE2T and 24 immune cells. **C** Heatmap of the correlation between UBE2T expression and various immune cell surface marker proteins: CR2 (B cells), CD8A (cytotoxic cells), SIGLEC8 (eosinophils), CD1A (iDCs), TPSAB1 (mast cells), B3GAT1 (NK cells), IL3RA (pDCs), CD3G (T cells), CD3D (T cells), CD3E (T cells), CD4 (T helper cells), PTPRC (Tcm), CXCR5 (Tfh), IL17A (Th17), GATA3 (Th2), and FOXP3 (Treg)

To determine the effect of UBE2T on the tumor microenvironment, immune infiltration analysis was performed using the ssGESA method. Spearman correlation analysis was used to calculate the correlation between immune cell enrichment and UBE2T expression in UCEC tissues. Results are shown in Fig. 5B, and the expression of UBE2T was positively correlated with the infiltration levels of Th2 cells, helper T cells, Tcm cells, and Tgd cells. The expression of UBE2T is associated with aDC, Th1 cells, DC cells, macrophages, CD8 T cells, B cells, Treg cells and NK CD56dim cells, Th17 cells, T cells, Tem cells, TFH cells, cytotoxic T cells, mast cells, NK cells, eosinophils, pDC cells, neutrophils, iDC cells, NK CD56bright cells showed a negative correlation with the infiltration levels of 20 immune infiltrating cells. Next, the expression profile data were divided into a high expression group and a low expression group according to the expression level of UBE2T to determine the changes in the levels of immune cell infiltration in different groups. The results shown in Fig. 5A showed that the infiltration levels of T helper cells, Th17 cells and Th2 cells in the low-expression group were significantly lower than those in the high-expression group. In addition, the infiltration levels of T cells, pDC, B cells, CD8 T cells, Cytotoxic cells, Eosinophils, iDC, Mast cells, Neutrophils, NK CD56bright cells, NK CD56dim cells, NK cells, Tem and TFH in the low expression group were significantly higher than those in the high expression group, consistent with the results shown in Fig. 5B. In order to verify the results of ssGESA, we analyzed the correlation between the expression of UBE2T and the expression of various surface marker proteins of immune cells and drew a heat map, as shown in Fig. 5C. The heat map shows a strong correlation between UBE2T and CR2, CD8A and FCGR3A, which is consistent with the results of the previous analysis.

### Clinical correlation analysis of UBE2T expression

**Fig. 6:**
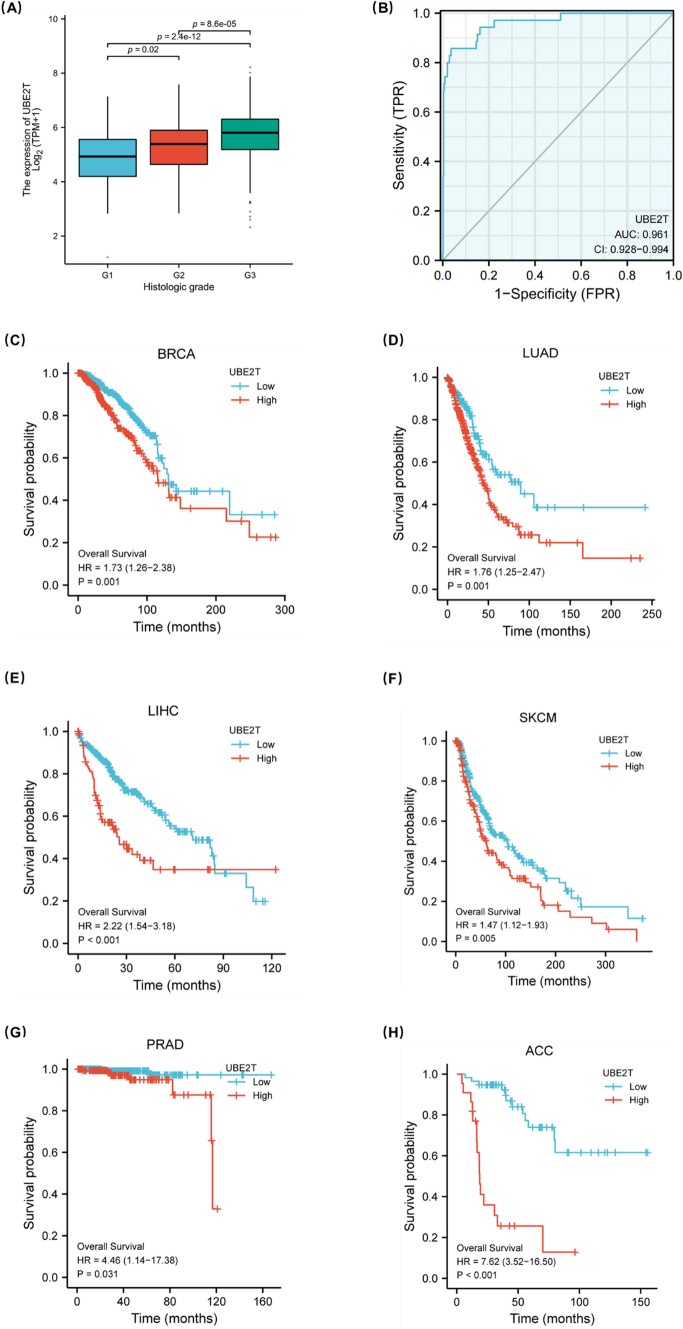
Clinical correlation analysis of UBE2T expression. The data from the TCGA database show that UBE2T is associated with prognosis. **A** Group comparison of UBE2T protein expression levels in samples with different histologic grades. **B** The diagnostic ROC curve of UBE2T. **C**–**H** Survival curves of the relationship between UBE2T Expression and OS in BRCA, LUAD, LIHC, SKCM, PRAD, ACC

In order to further verify that high expression of UBE2T is associated with poor prognosis and closely related to tumor development, we compared the expression levels of UBE2T in different groups of patients with different clinicopathological factors. As shown in Fig. 6A, there were significant differences in the expression levels of UBE2T among different histological grades (G1 vs G2, *p* = 0.02; G2 vs G3, *p* = 2.4e-12; G1 vs G3, *p* = 8.6e-05). It is suggested that the expression of UBE2T may be related to tumorigenesis and can be used as a marker for tumor diagnosis. In addition, correlation analysis showed that UBE2T expression has a certain prognostic value in various cancers. As shown in Fig. 6C–H, in breast cancer, lung adenocarcinoma, liver hepatocellular carcinoma, skin cutaneous melanoma, prostate adenocarcinoma and adrenocortical carcinoma, the survival time of the group with high expression of UBE2T was shorter than that of the group with low expression. As shown in Table 2, the clinical baseline data table was constructed according to the expression level of UBE2T for prognostic evaluation. The high expression of UBE2T is significantly associated with a higher histologic grade (particularly G3) and a higher rate of receiving radiation therapy. These results suggest that UBE2T may play a more important role in more aggressive endometrial cancer and could potentially influence treatment choices for patients. Finally, the ROC curve was constructed to verify the accuracy of UBE2T expression in predicting prognosis, as shown in Fig. B. The variable UBE2T had a sure accuracy in predicting the prognosis of tumor and normal patients (AUC=0.961, CI=0.928–0.994).

**Table 2:** Baseline data sheet from TCGA

Characteristic	Low expression of UBE2T	High expression of UBE2T	*p*
*n*	276	276	
*Clinical stage*, *n* (%)			0.962
Stage I	169 (30.6%)	173 (31.3%)	
Stage II	27 (4.9%)	24 (4.3%)	
Stage III	66 (12%)	64 (11.6%)	
Stage IV	14 (2.5%)	15 (2.7%)	
*Primary therapy outcome*, *n* (%)			0.328
PD	10 (2.1%)	10 (2.1%)	
SD	3 (0.6%)	3 (0.6%)	
PR	3 (0.6%)	9 (1.9%)	
CR	230 (47.9%)	212 (44.2%)	
*Race*, *n* (%)			0.776
Asian	9 (1.8%)	11 (2.2%)	
Black or African American	51 (10.1%)	57 (11.2%)	
White	191 (37.7%)	188 (37.1%)	
*Age*, *n* (%)			1.000
< = 60	103 (18.8%)	103 (18.8%)	
> 60	172 (31.3%)	171 (31.1%)	
*Weight*, *n* (%)			0.428
< = 80	116 (22%)	127 (24.1%)	
> 80	147 (27.8%)	138 (26.1%)	
*Height*, *n* (%)			0.695
< = 160	121 (23.1%)	126 (24.1%)	
> 160	141 (27%)	135 (25.8%)	
*BMI*, *n* (%)			0.344
< = 30	100 (19.3%)	112 (21.6%)	
> 30	159 (30.6%)	148 (28.5%)	
*Histological type*, *n* (%)			0.579
Endometrioid	200 (36.2%)	210 (38%)	
Mixed	12 (2.2%)	12 (2.2%)	
Serous	64 (11.6%)	54 (9.8%)	
*Residual tumor*, *n* (%)			0.815
R0	179 (43.3%)	196 (47.5%)	
R1	12 (2.9%)	10 (2.4%)	
R2	8 (1.9%)	8 (1.9%)	
*Histologic grade*, *n* (%)			** < 0.001**
G1	72 (13.3%)	26 (4.8%)	
G2	68 (12.6%)	52 (9.6%)	
G3	130 (24%)	193 (35.7%)	
*Tumor invasion* (%), *n* (%)			0.247
< 50	139 (29.3%)	120 (25.3%)	
> = 50	103 (21.7%)	112 (23.6%)	
*Menopause status*, *n* (%)			0.760
Pre	17 (3.4%)	18 (3.6%)	
Peri	10 (2%)	7 (1.4%)	
Post	227 (44.9%)	227 (44.9%)	
*Hormones therapy*, *n* (%)			1.000
No	143 (41.6%)	154 (44.8%)	
Yes	23 (6.7%)	24 (7%)	
*Diabetes*, *n* (%)			0.545
No	167 (37%)	161 (35.7%)	
Yes	58 (12.9%)	65 (14.4%)	
*Radiation therapy*, *n* (%)			**0.017**
No	155 (29.4%)	124 (23.5%)	
Yes	111 (21.1%)	137 (26%)	
*Surgical approach*, *n* (%)			0.713
Minimally Invasive	103 (19.4%)	105 (19.8%)	
open	166 (31.3%)	156 (29.4%)	
Age, median (IQR)	64 (57.5, 71.5)	64 (57, 71)	0.926

*CR* complete response, *PD* progressive disease, *PR* partial response, *SD* stable disease

### Prognostic analysis of UBE2T expression survival rate

**Fig. 7:**
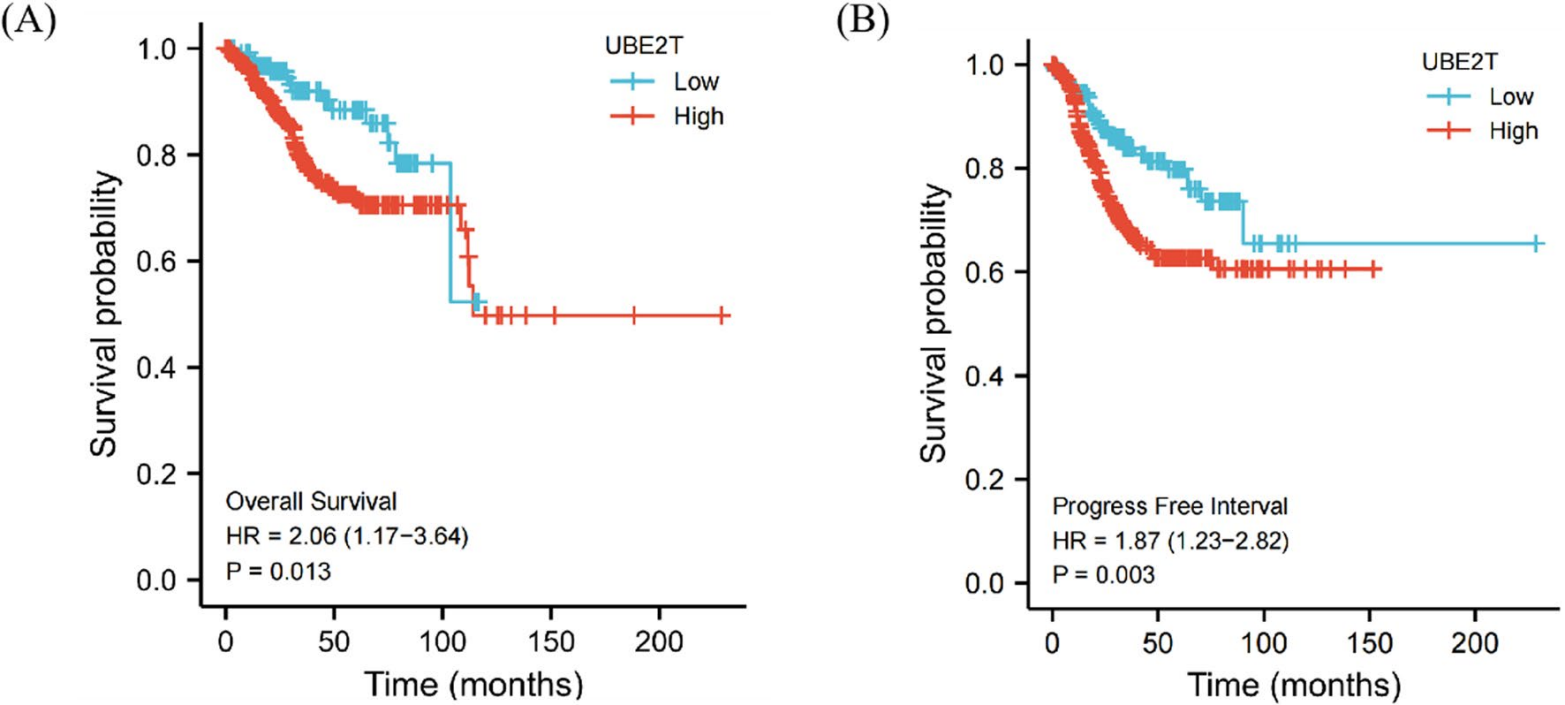
Prognostic analysis of UBE2T expression survival rate. The high expression of UBE2T is associated with poor prognosis. **A** Overall survival (OS). **B** Progression-free interval (PFI)

Firstly, the expression profile data were divided into a high expression group and a low expression group according to the expression level of UBE2T, and the overall survival (OS) and progression-free interval (PFI) of UCEC patients in different groups were analyzed. The results showed that the UBE2T high expression group had a shorter survival time, as shown in Fig. 7A and B. These results suggest that the high expression of UBE2T is associated with poor prognosis, suggesting that the elevated expression level of UBE2T is one of the risk factors for patient prognosis.

## Discussion

Endometrial cancer is one of the most common gynecologic malignancies in the female reproductive system, with its incidence showing an increasing trend year by year [26]. Although early-stage endometrial cancer patients can be cured through radical surgery, late-stage patients have higher rates of recurrence and mortality [4]. Therefore, early identification and intervention are crucial for improving patient prognosis. Screening for sensitive biomarkers helps detect UCEC (Endometrial Cancer) and formulate appropriate treatment plans [27].

This study utilized expression profile data of UCEC from TCGA and GEO databases to screen for differentially expressed genes between UCEC and normal tissues. It was found that UBE2T is highly expressed in UCEC compared to normal tissues, suggesting a potential association of UBE2T with the occurrence and development of UCEC. Similar to this study, Cao et al. [7] conducted a pan-cancer analysis of UBE2T and found that its high expression is a prognostic risk factor in most cancers, possibly serving as a therapeutic target or a predictive factor for prognosis and immunotherapy sensitivity. Further validation using collected clinical samples through immunohistochemical staining and protein imprinting analysis confirmed the high expression of UBE2T in UCEC, indicating that UBE2T could be a molecular biomarker for diagnosis and prognosis of UCEC patients.

To further elucidate the molecular mechanisms of UBE2T in the occurrence and development of endometrial cancer (UCEC), this study employed single-gene differential analysis and correlation analysis to construct a protein interaction network predicting UBE2T’s functions. It was found that 30 hub genes were most correlated with UBE2T expression, and 50 genes were closely associated with UBE2T expression. Notably, mitochondrial proteins showed a significant correlation with UBE2T expression. Mitochondrial gene overexpression is closely linked to cancer initiation and progression [28]. Research indicates that mitochondrial genes play crucial roles in energy metabolism, redox status, and cell apoptosis, potentially promoting tumor cell proliferation and metastasis when overexpressed [29]. Previous studies have suggested that targeting mitochondrial proteins could be a future strategy for cancer therapy [30], improving patient outcomes. Therefore, it is hypothesized that UBE2T might promote the occurrence and development of endometrial cancer through upregulating mitochondrial protein expression and enhancing mitochondrial function. Additionally, among the genes closely associated with UBE2T expression, genes like DTL, NUF2, and MELK may also contribute to the progression of endometrial cancer. Overexpression of DTL, for instance, can decrease PDCD4 expression levels, promote intracellular PDCD4 ubiquitination, accelerate tumor cell growth, and enhance invasive capabilities [31]. In comparison to normal tissues, NUF2 and CDCA2/3/4/5/8 are significantly upregulated in UCEC. Their biological functions primarily involve the cell cycle, DNA replication, base excision repair, mismatch repair, nucleotide excision repair, cellular senescence, and activation of the p53 signaling pathway [32]. MELK promotes endometrial cancer progression through activation of the mTOR signaling pathway [33]. These findings collectively suggest that UBE2T likely plays a crucial role in tumor initiation and development by participating in cell mitosis and the cell cycle.

To further elucidate the potential molecular mechanisms by which UBE2T promotes tumor development, this study conducted GO analysis, KEGG analysis, and GSEA analysis. GO analysis revealed that UBE2T is involved in biological processes such as keratinization, cornified envelope, and intermediate filament pathways. Research indicates that keratins are essential scaffold proteins in epithelial cells, regulating tumor cell apoptosis resistance, growth, and migration, and are closely associated with tumorigenesis [34, 35]. Elevated levels of certain keratins in serum or tumor tissues of cancer patients have been widely used for cancer diagnosis and are negatively correlated with patient prognosis, serving as prognostic indicators [36]. Intermediate filaments are known to inhibit UCEC metastasis and invasion [37], and UBE2T shows a negative correlation with pathways related to intermediate filaments. KEGG analysis indicated that UBE2T functions are associated with pathways such as cytochrome P450 metabolism, IL-17 signaling pathway, and retinol metabolism. High expression of cytochrome P450 can induce tumorigenesis and inactivate anticancer drug [38]. IL-17, as a potent pro-inflammatory cytokine, is closely associated with the formation, growth, and metastasis of various tumors, promoting tumor growth by activating proliferation-related pathways in endometrial cancer cell [39], such as NF-κB and STAT3. Although there is currently no direct evidence that clearly demonstrates a specific interaction between UBE2T and the IL-17 signaling pathway, considering the broad role of ubiquitination in signaling pathways, UBE2T may indirectly participate in the regulation of inflammatory responses and its role in cancer by modulating key components of the IL-17 signaling pathway. This hypothesis requires further experimental validation to reveal the specific functions of UBE2T within the IL-17 signaling pathway [40]. Retinol and its derivatives play roles in delaying or preventing precancerous lesions, inducing tumor cell differentiation and apoptosis [41]. GSEA analysis showed that UBE2T is closely associated with genes like K-Ras and NF-κB. K-Ras-related signaling pathways are persistently activated in many cancers, involved in cell growth, differentiation, protein synthesis, glucose metabolism, cell survival, and inflammation. NF-κB genes participate in tumor initiation and DNA repair processes, regulating the expression of cancer-related genes, and serving as prognostic indicators for cancer therapy [42]. In summary, the above analyses suggest that UBE2T is closely associated with tumor cell proliferation, necessitating further experimental research to validate the potential mechanisms by which UBE2T overexpression leads to poor prognosis in UCEC patients.

Next, this study analyzed the correlation between UBE2T and the infiltration of 24 immune cell types in UCEC tissues. The results showed that high UBE2T expression was negatively correlated with infiltration levels of T cells, pDC cells, B cells, CD8 T cells, cytotoxic cells, eosinophils, iDC cells, mast cells, neutrophils, and other immune cells. Low immune infiltration levels weaken the ability of the immune system to recognize and eliminate tumor cells, potentially leading to tumor growth and spread [43]. This is generally considered an unfavorable prognostic indicator, often associated with higher tumor stages and poorer survival rates. Additionally, Th2 cell infiltration was significantly positively correlated with UBE2T expression. In a healthy state, there is a dynamic balance between Th1 and Th2 cells, and an excessive shift towards one type may result in immune imbalance [44, 45]. Th2 cells dominate in the growth of various tumors such as non-small cell lung cancer, colorectal cancer, ovarian cancer, choriocarcinoma, melanoma, gastric cancer, osteosarcoma, lymphoma, and nasopharyngeal carcinoma, and their infiltration levels are positively correlated with the malignancy of tumors [46–48]. The study results suggest that upregulation of UBE2T may suppress the body’s anti-tumor immune response, making UBE2T a potential target for tumor immunotherapy. Further research in tumor microenvironment and experimental studies are needed to validate this possibility.

To explore the role of UBE2T in predicting patient prognosis, this study utilized TCGA database to analyze the relationship between UBE2T expression and clinical-pathological features. The analysis revealed a significant correlation between UBE2T expression and histological grade. Interestingly, immunohistochemical analysis of 113 clinical samples in this study showed that, in addition to histological grade, UBE2T protein expression levels were also associated with patient FIGO stage and menopausal status. Unlike transcript level analysis from RNA-seq databases, these clinical sample data were analyzed at the protein level using immunohistochemistry, which may introduce some differences in the analysis results. Future studies using proteomics and larger sample sizes are needed to validate the relationship between UBE2T protein expression levels and patient clinical characteristics. In survival analysis, patients with high UBE2T expression had significantly shorter overall survival (OS) and progression-free interval (PFI) compared to those with low UBE2T expression. The ROC curve showed an AUC of 0.961, indicating high accuracy of UBE2T in predicting patient prognosis. Therefore, UBE2T expression levels can effectively predict the prognosis of endometrial cancer patients.

In summary, increased expression of UBE2T in endometrial cancer tissues may suppress the anti-tumor immune response in UCEC patients, participating in various biological processes such as tumor initiation, metastasis, and invasion, and correlating with poorer survival rates. UBE2T holds potential as a prospective indicator for diagnosing and predicting prognosis in endometrial cancer patients. These findings pave the way for further research into the mechanistic role of UBE2T in endometrial carcinoma, offering new insights into potential clinical applications. However, there are limitations in this study that should be acknowledged. First, there is a significant imbalance between the number of normal samples and tumour samples, which may affect the robustness of the findings. Future studies with larger and more balanced sample sizes are required to validate the results. Additionally, the conclusions drawn from this study need further validation through comprehensive experimental approaches, including in vivo and in vitro models, to better elucidate the mechanism by which UBE2T promotes UCEC. Given that this is a retrospective analysis based on RNA sequencing data from the TCGA and GEO databases, the potential for inherent biases cannot be ruled out. Therefore, prospective studies are necessary to minimize these biases and provide stronger evidence for the role of UBE2T in UCEC.

## Data Availability

Publicly available datasets were analyzed in this study. This data can be found here: The UCSC XENA database (https://xenabrowser.net/datapages/), The TCGA database (https://portal.gdc.cancer.gov/), and GSE17025 of The GEO database (https://www.ncbi.nlm.nih.gov/geo/).
